# User satisfaction with train fares: A comparative analysis in five Australian cities

**DOI:** 10.1371/journal.pone.0199449

**Published:** 2018-06-21

**Authors:** Puteri Paramita, Zuduo Zheng, Md. Mazharul Haque, Simon Washington, Paul Hyland

**Affiliations:** 1 School of Civil Engineering & Built Environment, Science and Engineering Faculty, Queensland University of Technology, Brisbane, Queensland, Australia; 2 School of Civil Engineering, the University of Queensland, Brisbane, Queensland, Australia; 3 Business School, Queensland University of Technology, Brisbane, Queensland, Australia; University of Padua, ITALY

## Abstract

In the public transport industry, travellers’ perceived satisfaction is a key element in understanding their evaluation of, and loyalty to ridership. Despite its notable importance, studies of customer satisfaction are under-represented in the literature, and most previous studies are based on survey data collected from a single city only. This does not allow a comparison across different transport systems. To address this underrepresentation, this paper reports on a study of train passengers’ satisfaction with the fare paid for their most recent home-based train trip in five Australian capital cities: Sydney, Melbourne, Brisbane, Adelaide, and Perth. Two data sources are used: a nation-wide survey, and objective information on the train fare structure in each of the targeted cities. In particular, satisfaction with train fares is modelled as a function of socio-economic factors and train trip characteristics, using a random parameters ordered Logit model that accounts for unobserved heterogeneity in the population. Results indicate that gender, city of origin, transport mode from home to the train station, eligibility for either student or senior concession fare, one-way cost, and waiting time as well as five diverse interaction variables between city of origin and socio-economic factors are the key determinants of passenger satisfaction with train fares. In particular, this study reveals that female respondents tend to be less satisfied with their train fare than their male counterparts. Interestingly, respondents who take the bus to the train station tend to feel more satisfied with their fare compared with the rest of the respondents. In addition, notable heterogeneity is detected across respondents’ perceived satisfaction with train fare, specifically with regard to the one-way cost and the waiting time incurred. An intercity comparison reveals that a city’s train fare structure also affects a traveller’s perceived satisfaction with their train fare. The findings of this research are significant for both policy makers and transport operators, allowing them to understand traveller behaviours, and to subsequently formulate effective transit policies.

## Introduction

Satisfaction is an essential concept in the service industry and market research, and an important factor in understanding customer behaviour [1–7]. In public transport, user satisfaction is recognized as a key link between public transport provider offerings and traveller reactions to these offerings [8]. In general, knowledge of the level of user satisfaction with the existing public transport system provides valuable information for both policy makers and public transport operators, providing the basis for the development of effective strategies for improving traveller experience and, in turn, increasing ridership.

Only a few studies in the literature focus on user satisfaction in the context of public transport [8–11]. Available studies identify that frequency, reliability, driver behaviour, information, cleanliness, and comfort are typical factors that influence public transport users’ satisfaction with services [2, 12–14]. Despite its enormous importance, user satisfaction studies are generally under-represented in the literature. In addition, the fact that most previous studies are based on survey data collected from a single city makes a comparative analysis across different public transport systems almost impossible. In addition, the data used in many previous studies only partially captures the potential factors that could be significantly linked to user satisfaction. An inter-city comparison supplemented by information on the characteristics of the current transport system in each city can be valuable in revealing underlying (or even causal) factors that contribute to user satisfaction with public transport services.

To control confounding factors, this study specifically focuses on train riders’ satisfaction with the paid train fare for their most recent home-based train trip in five Australian capital cities: Sydney, Melbourne, Brisbane, Adelaide, and Perth. It uses two main data sources: a nation-wide survey, and objective information on the characteristics of each city’s existing train system. An ordered Logit model is developed to identify significant factors associated with train user satisfaction levels. To gain more insights, underlying reasons behind differences in user satisfaction across the five cities are traced back to relevant characteristics of the existing train systems (and to fare structure in particular) in these cities. Findings of this study can be used by policy makers to develop effective strategies for enhancing train rider satisfaction, and to increase train patronage as a result.

The remainder of the paper is structured as follows: Literature review section presents a comprehensive, related literature review; the section ‘context: train fare structures in the five cities’ provides the study context and information on the train fare structure in each of the targeted cities, which is the base knowledge for analysing the nation-wide survey data; Dataset section describes the data source and descriptive analysis; Methodology section introduces the modelling methodology; Results section discusses the results of the modelling analysis; Intercity comparison section focuses on the inter-city comparison, and links characteristics of the existing train systems in these cities to the modelling results and finally, Conclusions section summarises the main conclusions of the study and discusses topics for future research.

## Literature review

This section reviews notable studies on user satisfaction with public transport. User satisfaction is a multi-dimensional concept [7]. Parasuraman et al.[15, 16] and Zeithaml et al. [17] identified five general dimensions of user satisfaction, including assurance, reliability, empathy, tangibles, and responsiveness. Friman et al.[18] proposed a model to assess user satisfaction based on simplicity of design and information, treatment by staff, and service reliability. As a slightly different concept, a service quality model of user satisfaction consisting of functional and technical service attributes was proposed by Grönroos [19, 20]. Technical service attributes are related to what services the customer receives, while functional service attributes are related to how the customer receives those services. Later, Fellesson and Friman [8] labelled these two attributes ‘Factor A’ and ‘Factor B’, with Factor A being a safety factor related to feeling secure at stations and on-board vehicles, and Factor B being a system factor related to frequency, and travel and waiting times. In addition, Fellesson and Friman [8] defined three other factors: Factor C, related to public transport comfort (e.g., in-vehicle cleanliness and level of crowding); Factor D related to the behaviour, knowledge, and attitude of the staff; and Factor E related to service delivery.

Satisfaction is also considered to be the foundation of consumer loyalty and behaviour [21, 22], and has a strong connection with perceived value and service quality [2–5, 23–25]. Travellers who have experienced a good quality public transport services are incline to have a higher level of perceived satisfaction, and intend to continue using the same services. Satisfaction is also related to a traveller’s evaluation of the quality of their entire trip experience [1, 23, 26, 27].

Beirão and Cabral [13], Gadziński and Radzimski [28], Tyrinopoulos and Antoniou [29], and Efthymiou, Antoniou, and Tyrinopoulos [9] analysed the behaviour of public transport travellers and their perceived satisfaction with services in order to understand the underlying reasons for their preferred transport mode. They found that travellers had a strong preference for a reliable and well-coordinated transportation system. The cleanliness of vehicles, the condition of the waiting area, service frequency, network coverage, and transfer distance were perceived as the most important satisfaction attributes. This knowledge served as a foundation for policy makers and transport operators to improve services by making operational adjustments to frequency of services, transfer points, and network coverage [29, 30].

Travellers who have experienced unreliable services and long waiting time have a low public transport user satisfaction level [31, 32]. The importance of travel time reliability was influenced by two factors: negative consequences for travellers arriving late at their destinations, and the actual value that individual travellers placed on reliability of transportation system [26, 33]. Lucas and Heady [34] discussed the concept of time urgency and assessed the difference between travellers’ with a flexitime schedule and those without such flexibility. Time urgency was found to be a personal concept relating to the individual’s perception of time. Since flexitime scheduling greatly reduced commuting pressure, they argued that flexitime travellers experienced less time urgency and more trip satisfaction[35].

Another essential factor in enhancing public transport service quality is the reduction of on-board crowding [31]. On-board crowding positively contributes to the perceived walking and waiting time [36]. In reality, travel time is actually longer when vehicles are crowded because it takes longer to board and alight when there are passengers standing in aisles. Li and Hensher [36] argued that a successful public transport system requires less crowding and increased reliability at all phases of the service chain. Reliability is defined as the quality of performing consistently well.

The availability of pre-departure travel information is vital for travellers, and eventually affects their travel habits and satisfaction levels [37]. Pre-departure travel information has a number of roles, such as providing an awareness of the travel options for a particular trip; empowering travellers to make fully informed travel choices; assisting travellers to successfully commence and complete a trip; and reducing waiting travellers’ feelings of frustration stress [31, 37–40]. The success of such information, however, depends on its reliability and its accessibility by all travellers [41]. Regular travellers formally obtain such information when they first use a particular service, while occasional travellers rely on informal information from others [37].

With particular reference to train services, Brons et al. [30] mentioned a number of important factors that affect travellers’ perceived satisfaction: station organization, real-time information, level of comfort, service punctuality, train frequency, and accessibility. Accessibility includes bicycle parking, connections to the wider public transport network, and car parking facilities at stations. Furthermore, in order to increase ridership, Ellaway et al. [42] and Brons et al. [30] suggested the provision of benefits similar to those enjoyed travelling by private transport. Examples include the integration of cycling and train travel by permitting bicycles on board; the provision of bike hooks on certain trains during off-peak hours; and the provision of park-and-ride facilities at major stations to entice motorists to use the train as a part of their trips [43, 44].

In addition, individual characteristics such as age, income, education level, household composition, travel budget, a drivers’ license, and access to a motor vehicle affect mobility behaviour and the level of access to train services [9, 45–47]. Specifically, in order to attract potential train riders and increase ridership, train services should be designed according to the level of service preferred by current riders [13].

Overall, the majority of the previous studies focussed on users’ overall satisfaction, and only a few investigated user satisfaction with train fare [26]. This is despite the fact that train fares, as the only monetary cost incurred by train riders, is often used as a powerful tool to influence ridership. Thus, our understanding of the relationship between train fare and user experience remains elusive. In addition, most of the previous studies were based on survey data collected from a single city, thus precluding the possibility of intercity comparison. Hence, this comparative analysis study, which is complemented by knowledge of the characteristics of the current fare system in each city, is valuable in revealing the underlying and causal factors that contribute to travellers’ perceived satisfaction. The data used in many previous studies only partially capture the potentially significant factors linked to traveller satisfaction. In particular, characteristics of travellers’ most recent trips are often not considered.

## Context: Train fare structures in the five Australian cities

A city’s geographical spread, land-use planning, and overall public transport network also influence travellers’ perceptions of the overall transportation system, and especially their perceived satisfaction with public transport services [48]. In the data analysis of this study, objective information on the characteristics of the existing train fare structure in each of five Australian capital cities was used to explain the diverse perceived satisfaction with train fare. Intuitively, information on the current train fare structure in each of the targeted cities is the base knowledge for analysing the nation-wide survey data. Particularly, such information can assist readers in better understanding underlying reasons behind the differences in user satisfaction across the five cities revealed by our modelling analysis. Thus, the current train fare structure in each city is explained in this section.

### Sydney

“Opal” is a smartcard system that has been implemented by Transport for New South Wales for commuters using various public transport modes across Sydney, the Blue Mountains, Central Coast, Hunter or the Illawarra region [49]. The correct amount of fare is automatically deducted, and is based on the distance travelled. Four different fares are available: Adult, Child/Youth, Concession and Senior/Pensioner. With respect to train services, it costs $3.38 for an adult, $ 1.69 for a child, $ 1.69 for concession-eligible travellers, and $ 1.69 for seniors/pensioners to travel between 0 to 10 km; and up to $8.30 for an adult, $ 4.15 for a child, $ 4.15 for concession-eligible travellers, and $ 2.50 for seniors/pensioners to travel above 65 km one-way during peak hours.

The off-peak fare is 70% of the peak fare. The off-peak periods are outside the peak periods of 7:00 to 9:00 AM and 4:00 to 6:30 PM for Sydney train services, and outside the peak periods of 6:00 to 8:00 AM and 4:00 to 6:30 PM for intercity train services. There is a $ 2.50 cap for all Opal trips taken on Sundays, and a weekly travel reward for travellers who make eight paid trips in a week [49]. Travellers are entitled to save around 20% by using their Opal card rather than purchasing single trip tickets.

### Melbourne

Public Transport Victoria [50] issues “Myki”, a smart card, as its method of payment across public transport in Melbourne. This card automatically deducts the lowest fare possible, based on the travellers’ departing and alighting locations. The card is divided into two categories, Myki money and Myki pass. Full and concession fare are available for each category. There is a cap of $6 for a day fare during the weekend and public holiday when using Myki across Zones 1 and 2 [50]. Travellers who have touched off before 7:15 AM are eligible for a free early bird train travel. Two fare bands, Zones 1+2 and Zone 2 are available for Myki money for both 2-hour usage and daily fare. Zone 1 of Melbourne’s train, tram, and bus networks is the CBD and inner city suburbs, an approximately 12 km radius. Zone 2 covers the middle and outer suburbs.

### Brisbane

A smartcard system, “Go Card”, has been implemented by Translink in the South East Queensland Region, including Brisbane. Go Card allows travellers to travel seamlessly on all public transport; i.e., on Translink’s bus, ferry, train, and tram services [51]. Travellers are entitled to savings when they use a Go Card rather than paper tickets. The Go Card automatically calculates and deducts the overall fare at either an adult or concession rate, based on the number of zones travelled through the trip [51].

The off-peak Go Card fares are 80% of the peak fares. The off-peak periods are between 8:30 AM and 3:30 PM and 7:00 PM and 6:00 AM on weekdays, and all weekend. Paper tickets cost 130% of the Go Card fare, while the concession fares are 50% of adult fares [51].

### Perth

Perth public transport has two types of ticket: a SmartRider card and cash tickets. The SmartRider card is a refillable smart card with seven categories: standard, concession, senior, pensioner, veteran, student, and tertiary [52].

With the exception of two-section fares (valid for a single one-way trip), cash tickets have an expiry time; however, within the allowable time travellers are free to ride on any number of buses, trains, and ferry services to complete their trip [52]. Travellers who use SmartRider should remember to touch on and off upon boarding and alighting from public transport services. The expiry times are two hours for a trip of one to four zones, and three hours for a trip of five or more zones. Overall, Transperth (public transport system serving the city and suburban areas of Perth) covers services across nine different zones. Each zone has an approximately 8 km radius. Within the city centre, there is a free zone area, where all public transport is completely free.

### Adelaide

There are two public transport tickets available in Adelaide, “Metrotickets” and “Metrocard” [53]. Metrotickets are paper tickets for both single and day trips across Adelaide Metro trains, trams, and buses, and are the best option for infrequent public transport users. Metrocard, on the other hand, is an electronic smart card designed for multiple public transport trips on Adelaide Metro trains, trams, and buses.

There is no public transport zone division in Adelaide; therefore, fares are not calculated according to the distance travelled. Overall, there are four different fares offered to Adelaide public transport users: regular fares, concession and tertiary student fares, primary and secondary student fares, and senior Metrocard [53]. The interpeak, regular Metrocard fares are 55% - 75% of the regular peak fares. The peak periods are before 9:01 AM and after 3:00 PM on weekdays, and all day Saturday. Inter peak periods are Monday to Friday 9:01 AM to 3:00 PM, and all day Sundays and public holidays. Concession and tertiary student as well as senior Metrocard fares are 50% of regular Metrocard fares, while primary and secondary student Metrocard fares are 30% of regular Metroard fares.

## Dataset

### Data collection

The data used in this research are the online survey data collected by the project team of the “CRC for Rail Innovation Project 1.130: Urban Rail Travel Behaviour” [54]. The team’s survey questionnaire was designed with four primary objectives: to identify what factors drive the patronage of urban trains; to quantify the relative importance of these patronage drivers; to allow national comparisons of satisfaction with train usage in five Australian cities; and to develop a national database as a resource for further research. In order to efficiently achieve these objectives, the project team synthesized the earlier published best practice surveys from Australia and overseas, identified the gaps in this work, and then addressed these gaps [54]. Details on how the questionnaire was designed and tested can be found in Zheng et al. [54], and Zheng et al.[55].

The survey was conducted via a web-based survey platform in five Australian cities from 9 April 2013 to 16 May 2013. Seven thousand respondents were targeted, 2,000 in both Sydney and Melbourne, and 1,000 in each of the remaining cities (i.e., Brisbane, Adelaide, and Perth). The target number for each city was proportional to its population. Half of the respondents from each city were expected to be train riders, and the other, non-riders. The train rider group in this study is defined as ‘respondents who made more than one trip by train in the month prior to the survey’, while the other group is defined as ‘non-riders’ [54]. Eventually, a total of 6,731 respondents participated in the survey. Of these, 3,231 are train riders: 1,000 from Sydney; 1,000 from Melbourne; 489 from Brisbane; 242 from Adelaide; and 500 from Perth. With the exception of the Adelaide train rider group, all targeted quotas were achieved [54].

Census data from the Australian Bureau of Statistics (ABS) were used to monitor gender and age of the respondents to ensure that the sample was representative of Australian cities. Age and gender groups obtained were close to those in the ABS data, thus confirming the sample as representative. For more information on the survey, see [54, 55].

### Data preparation

To ensure the quality of the survey data, several factors were checked for outliers, including one-way train fare, travel time, waiting time, and access time. Respondents were asked to provide the amount of fare they paid for their most recent train trip. As a commonly used outlier detection method, train fares outside the range of Q1–3* (Q3-Q1) ($ 0) to Q3 + 3* (Q3-Q1) ($ 17.6) were regarded as potential outliers, where Q3 and Q1 were the third and first quantile, respectively [56, 57]. The same procedure was then applied to on-board time, and any on-board time outside the range of 0 to 135 minutes was regarded as a potential outlier. Similarly, waiting time outside the range of 0 to 25 minutes was regarded as a potential outlier, and any total access time outside the range of 0 to 75 minutes was also regarded as a potential outlier.

Prior to removing any outliers, the precaution of seeking additional information to confirm their outlier status was taken. More specifically, by checking the actual train fare structures in the five cities, this study found that the maximum train fare for a one-way trip of around 135 minutes is $28. Thus, $28 was used as the upper fare limit. After this data cleansing, 2,927 respondents (out of 3,231) were included in the detailed analysis.

Correlation analysis was performed prior to modelling in order to obtain valuable knowledge about potential relationships among explanatory variables. More specifically, Pearson correlation analysis was performed for the continuous variables, while Spearman correlation analysis was performed for the categorical and ordinal variables [58, 59]. It was found that i) total travel time has a significant positive correlation with on-board time, waiting time, and total access time; and that ii) age group has significant correlations with the type of concession fare. Therefore, total travel time and age group are not included in the model. In addition, paid fare status and departure time are strongly correlated because paid fare status is derived from departure time. Thus, departure time is also not included in the model.

### Data description

Each train rider’s socio-economic profile and the characteristics of their most recent train trip were collected in the survey. Socio-economic background includes information about their gender, age, employment status, pre-tax household weekly income level, highest education level, whether they hold a driver’s license, whether they have access to a vehicle, whether a motor vehicle is required for their work, whether train services influenced the choice of their current home location, and the composition of their household. Meanwhile, characteristics of their most recent train trip include departure time, the purpose of their trip, pre-departure information check, home-to-station transport mode, whether there was any on-board crowding or on-board activities, transport mode from the station to their destination, the one-way trip cost, the time spent on-board, waiting time, total access time, and total travel time.

In the five cities chosen for this study, at least two types of concessions fare are available: a senior concession fare, and a student concession fare. However, respondents were not directly questioned about these fares in the survey. Given the eligibility requirements for concession fares set by transport authorities, the following assumptions were made in order to accurately understand train riders’ satisfaction with their fare: (a) A respondent who is at least 60 years old and is retired is eligible for a senior concession fare; and (b) A respondent who is between 16 and 30 years old and is a student is eligible for a student concession fare [60, 61]. The respondents who do not belong to either of the concession groups pay the full adult fare. Meanwhile, train fares can differ depending on the travelling period. Respondents who departed from home between 7:00 PM and 7:00 AM and between 9:00 AM and 3:00PM are assumed to pay a discounted fare, and those who departed between 7:00 and 9:00 AM and between 3:00 and 7:00 PM are assumed to pay full fare.

**Fig 1:**
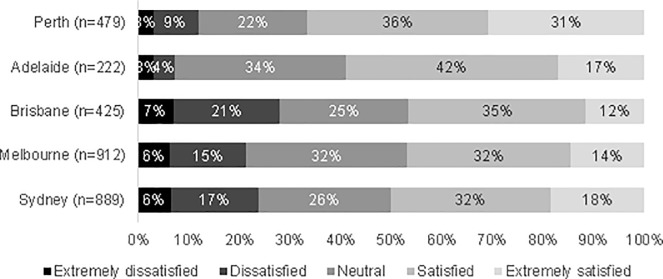
Satisfaction with train fare in each city.

The respondents were also asked to rate their perceived satisfaction with the train fare for their most recent home-based train trip, using a 5-point Likert scale; i.e., extremely satisfied, satisfied, neutral, dissatisfied, and extremely dissatisfied. Fig 1 shows the perceived satisfaction in each city. About 46% to 50% of respondents from Sydney, Melbourne, and Brisbane were satisfied or extremely satisfied with the fare paid for their most recent train trip, while less than 28% of respondents from each of those cities were not satisfied or extremely dissatisfied. Over 59% of respondents of Adelaide and Perth were satisfied or extremely satisfied with the fare paid for their most recent train trip, and less than 12% of respondents from each of those two cities were not satisfied or extremely dissatisfied.

The train riders’ socio-economic background and their most recent train trip characteristics are summarised in Tables 1 and 2, respectively.

**Table 1:** Socio-economic profiles of respondents from each city.

Socio-economic factor	Classification	Sydney (n = 889)	Melbourne (n = 912)	Brisbane (n = 425)	Adelaide (n = 222)	Perth (n = 479)
Gender	Male	48%	49%	40%	51%	41%
	Female	52%	51%	60%	49%	59%
Age group	Under 31 years old	22%	25%	20%	23%	21%
	Between 31 and 60 years old	62%	62%	63%	63%	55%
	Above 60 years old	16%	14%	17%	14%	24%
Employment status	Full-time	50%	48%	44%	37%	35%
	Part-time	18%	17%	16%	17%	19%
	Self-employed	6%	6%	6%	3%	5%
	Outside work force^a^	27%	29%	34%	43%	41%
Pre-tax household weekly income level	Low income	23%	25%	29%	39%	35%
	Middle income	35%	35%	36%	34%	27%
	High income	23%	21%	21%	8%	18%
	Not reported	19%	20%	14%	19%	20%
Highest education level	Pre-bachelor degree	46%	44%	52%	60%	56%
	Bachelor degree level	27%	29%	24%	24%	26%
	Graduate level and above	27%	27%	24%	16%	19%
Driver license	Yes	88%	88%	89%	83%	86%
	No	12%	12%	11%	17%	14%
Vehicle access	No access and solely dependent on public transport	20%	17%	17%	19%	18%
	Access to either privately owned, company owned, or shared motor vehicles	80%	83%	83%	81%	82%
Whether a motor vehicle is required for work	Yes	21%	25%	25%	23%	25%
	No	79%	75%	75%	77%	75%
Train services’ influence on the current home location decision	Significant	49%	44%	39%	24%	36%
	Moderate	21%	24%	24%	26%	18%
	Insignificant	29%	32%	37%	50%	46%
Household composition	Adults and children	36%	37%	35%	40%	33%
	Adults only	48%	46%	48%	47%	50%
	Multiple-family	16%	17%	17%	13%	17%

^a^”Outside work force” employment status includes being student, retired, on maternity or on other official working leaves, and being unemployed.

**Table 2:** Trip characteristics of train riders from each city.

Trip characteristic	Classification	Sydney (n = 889)	Melbourne (n = 912)	Brisbane (n = 425)	Adelaide (n = 222)	Perth (n = 479)
Departure time	Off-peak	50%	52%	51%	58%	57%
	AM peak	42%	34%	37%	33%	31%
	PM peak	8%	14%	12%	9%	12%
Trip purpose	Work/School	69%	66%	62%	58%	57%
	Social/ Recreational	7%	10%	7%	12%	14%
	Shopping/ Personal Business	25%	24%	31%	30%	29%
Pre-departure information check	Yes	47%	49%	51%	54%	37%
	No	53%	51%	49%	46%	63%
Transport mode from home to the train station	Bus	19%	16%	15%	18%	31%
	Walking	52%	43%	33%	45%	23%
	Driving	20%	29%	40%	25%	32%
	Cycling	0%	1%	0%	1%	2%
	Dropped-off	8%	11%	13%	12%	12%
On-board crowding^a^	Not crowded	82%	82%	88%	92%	74%
	Crowded	11%	8%	6%	4%	10%
	Overcrowded	7%	11%	6%	4%	16%
On-board activities	Work/study	8%	5%	3%	4%	2%
	Leisure	92%	95%	97%	96%	98%
Transport mode from the train station to the destination	Bus/tram	93%	83%	92%	87%	91%
	Walking	2%	2%	5%	4%	4%
	Picked up	1%	14%	1%	8%	2%
	Cycling	3%	2%	3%	2%	4%
Concession fare	Student concession fare	5%	5%	4%	10%	6%
	Senior concession fare	9%	8%	13%	11%	16%
	No concession (Adult) fare	86%	87%	84%	79%	78%
Paid fare status	Full Fare	50%	48%	49%	42%	43%
	Discounted Fare	50%	52%	51%	58%	57%
One-way cost ($)	Minimum	0	0	0	0	0
	Median	2.5	1.9	2.95	1.5	0.97
	Maximum	25.8	20	26	25	25.5
	Mean	3.511	2.460	3.319	2.022	1.891
	Standard deviation	3.535	2.881	4.038	2.627	2.667
On-board time (Minutes)	Minimum	1	1	3	1	1
	Median	30	30	30	25.5	20
	Maximum	135	130	135	120	90
	Mean	32.285	31.912	33.904	31.059	24.282
	Standard deviation	20.287	18.316	21.040	17.358	16.055
Waiting time (Minutes)	Minimum	1	1	1	1	1
	Median	8	7	10	10	7
	Maximum	25	25	25	25	25
	Mean	8.351	8.231	9.299	8.824	7.816
	Standard deviation	4.861	4.911	5.008	4.966	4.435
Total access time^b^ (Minutes)	Minimum	1	3	1	1	2
	Median	20	20	20	25	20
	Maximum	75	75	75	75	67
	Mean	23.172	23.113	22.758	26.752	22.132
	Standard deviation	13.570	13.945	13.452	15.399	13.171
Total travel time (Minutes)	Minimum	8	8	8	3	14
	Median	60	59	60	63.5	50
	Maximum	180	194	185	165	160
	Mean	63.808	63.257	65.960	66.635	54.230
	Standard deviation	25.962	24.876	28.284	26.053	23.189

^a^The on-board crowding is self-reported with three pre-defined categories: not crowded (a traveller found a seat for the entire journey); crowded (a traveller had to stand up prior to finding a vacant seat on-board); and overcrowded (a traveller had to stand up for the entire journey and no vacant seat available) [31].

^b^Total access time is defined as the total of the time a respondent spent travelling between their point of origin and the train station and the time they spent travelling between the train station and their destination.

## Methodology

This survey has Southern Cross University ethics approval number ECN-12-307. A consent form was provided to each respondent at the first page of the questionnaire. The survey was hosted by ORU (The Online Research Unit: http://www.theoru.com/) utilising their ‘research only’ online consumer panels. The resources provided access to more than 300,000 profiled persons. This also allowed structured data collection method that provided representative sampling from each city.

The satisfaction with train fare for the most recent train trip from home was ordered and categorical. In this analysis, satisfaction was treated as ordinal rather than nominal to provide simpler interpretations, greater flexibility, greater detection power, and more similarity to ordinary regression analysis [9, 62]. In this study, travellers’ satisfaction with train fare was estimated as a function of a number of socio-economic factors and trip characteristics, using an ordered Logit model. The Logit model used the cumulative distribution function of the logistic distribution [63]. The Logit model can be generalized to account for non-constant error variances in more advanced econometric settings, such as heteroskedastic or random-parameter Logit model. Constraining all parameters to be fixed while they are actually heterogeneous could lead to biased, inefficient, and inconsistent parameter estimates [64, 65].

In the context of this study, heterogeneity could arise in many factors such as on-board crowding, one-way cost, on-board time, waiting time, total access time, and total travel time [4, 13, 30, 40, 55, 65]. To capture such heterogeneity, these factors were considered as random parameters in the model development because different respondents might perceive them differently. For instance, a $2 one-way cost and 15 minutes waiting time can be perceived by one user as a cheap fare, and a short waiting time; however, another user could consider the fare to be expensive and the waiting time to be long. Such heterogeneity is influenced by individuals’ various socio-economic factors and trip experience.

The formulation of the ordinal data modelling problem, which is motivated by the latent regression perspective, is defined as

$$Y=j\;if\;\alpha_{j-1}<Y^{*}\leq \alpha_{j}$$ (1)

Y* is a continuous latent variable and is assumed to underlie the observed ordinal data. Particularly, Y* = *β*′*X* + *ε* and X is a vector of explanatory variables, *β* is a vector of coefficients, and *ε* is an error term. While, *j* is an ordinal responses and *α* is a set of cut points of the continuous scale for Y* [62, 66]. *Y* is observed to be in category j when the latent variable falls in the *j*^*th*^ interval.

In order to maintain the order of ordinal dependent variables, the logit transformation is applied to the cumulative probabilities, as below

$$logit[P(Y_{i}\leq j)]=\log\left(\frac{P(Y_{i}\leq j)}{1-P(Y_{i}\leq j)}\right)$$ (2)

A general model for the cumulative logits is shown below

$$logit\;[P(Y_{i}\leq j)]=\beta_{1}X_{1}+\beta_{2}X_{2}+\cdots +\beta_{n}X_{n}+\varepsilon_{i}=\beta \prime X+\varepsilon_{i}$$ (3)

,where *j* = 1,…,*c* − 1; *c* is the total number of categories. *X*_1_,*X*_2_,…,*X*_*n*_ are the *n* explanatory variables; *β*_1_,*β*_2_,…,*β*_*n*_ are the corresponding coefficients [62]. In this study, *Y*_*i*_ denotes the perceived satisfaction with the train fare of *i*^*th*^ respondent.

In the fixed parameter ordered Logit model above, the vector of parameters *β* is the same for all observations. On the other hand, a random-parameter ordered Logit model explicitly accounts for heterogeneities by allowing the regression coefficients to vary across observations [66, 67], as shown in Eq 4

$$\beta_{i}=\beta +u_{i}$$ (4)

,where *β*_*i*_ is a vector of random regression coefficients and *u*_*i*_ is a vector of randomly distributed terms for each regression coefficient. The additional error term *u*_*i*_ is correlated with the error term *ε*_*i*_ of the perceived satisfaction function, and thus translates individual heterogeneities into parameter heterogeneities. From Eqs 3 and 4, the function for the perceived satisfaction level becomes

$$logit\;[P(Y_{i}\leq j)]=\beta_{i}\prime X+\varepsilon_{i}=\beta^{\prime }\;X_{i}+{(u}_{i}^{\prime }\;X_{i}+\varepsilon_{i})$$ (5)

In this study, coefficients of on-board crowding, one-way cost, on-board time, waiting time, total access time, and total travel time variables are considered as candidate random-parameters. More specifically, each of the random parameters is assumed to follow a lognormal distribution restricted to the negative side because the expected sign of the estimates is known to be negative.

A simulation-based maximum likelihood method is employed to estimate the random-parameter ordered Logit model. Halton sequence (that provides more accurate approximations for numerical integrations than purely random draws) is used to obtain the simulation-based estimation [68–70]. Following the recommendation in the literature, 500 random Halton draws are used in estimating the random-parameters [68, 71, 72]. Following the standard model development process, the best fixed- and the random-parameter models are obtained based on the statistical significance independent variables and the Akaike’s Information Criteria (AIC) [73].

In the case of availability of two points from the explanatory variables, *X*_*a*_ and *X*_*b*_, then the cumulative logit is defined as

$$logit\;[P(Y_{i}\leq j|X_{a})]-logit\;[P(Y_{i}\leq j|X_{b})]=\beta^{\prime }\;(X_{a}-X_{b})$$ (6)

Eq 6 specifies that the odds of making response *Y* ≤ *j* at *X*_*a*_ are *exp*(*β*′ * (*X*_*a*_ − *X*_*b*_)) times the odds of *X*_*b*_. The log odds ratio is proportional to the distance between these two points, and the proportionality remains constant across different categories. Hence, Eq 3 is referred to as a “proportional odds” model. Due to its easy interpretation, this model has been widely used in the literature [62, 66, 74, 75].

## Results

Table 3 shows the summary of the best fixed-parameter and random-parameter ordered Logit models of perceived satisfaction with train fare. A likelihood ratio test was used to compare the performance of these models; the result shows that in terms of explaining travellers’ perceived satisfaction with their train fare, the random-parameter ordered Logit model performs statistically better than the fixed-parameter model, at a 95% significance level.

**Table 3:** Summary of the best fixed-parameter and random-parameter Logit models.

Explanatory Variables	Fixed-parameter model			Random-parameter model		
	Coefficient	z-statistics	p-value	Coefficient	z-statistics	p-value
Constant	3.684	25.18	<0.05	4.349	22.14	<0.05
Female	-0.255	-3.69	<0.05	-0.293	-4.15	<0.05
Sydney	-0.328	-2.29	<0.05	-0.357	-2.14	<0.05
Melbourne	-0.371	-2.75	<0.05	-0.419	-2.63	<0.05
Brisbane	-0.478	-3.12	<0.05	-0.552	-3.16	<0.05
Perth	0.406	2.55	<0.05	0.398	2.22	<0.05
Employment status: Outside work force	0.205	1.91	0.057	0.118	1.07	0.284
Transport mode from home to the train station: Bus	0.233	2.32	<0.05	0.404	3.83	<0.05
Student concession fare	-0.488	-2.56	<0.05	-0.435	-2.16	<0.05
Senior concession fare	2.349	11.4	<0.05	2.448	11.70	<0.05
Sydney * Employment status: Outside work force	0.460	2.35	<0.05	0.608	3.11	<0.05
Perth * Transport mode from home to the train station: Bus	-0.438	-2.06	<0.05	-0.491	-2.23	<0.05
Sydney * Student concession fare	-0.856	-2.35	<0.05	-1.024	-2.74	<0.05
Melbourne * Senior concession fare	-1.288	-4.36	<0.05	-1.350	-4.31	<0.05
Brisbane * Senior concession fare	-1.155	-3.48	<0.05	-1.077	-2.95	<0.05
One-way cost ($)^a^	0.082	7.37	<0.05	-2.482	-16.49	<0.05
Waiting time (Minutes)^a^	0.038	5.29	<0.05	-2.988	-16.51	<0.05
μ_1_^b^	1.514	35.80	<0.05	1.848	20.51	<0.05
μ_2_^b^	2.917	72.84	<0.05	3.404	33.90	<0.05
μ_3_^b^	4.781	84.09	<0.05	5.354	47.48	<0.05
Log-likelihood at convergence	-4024.145			-3976.970		
AIC	2.763			2.739		

^a^random parameters

^b^μ_1_, μ_2_ and μ_3_ are the thresholds of perceived satisfaction with train fare estimated by the model.

The random-parameter ordered Logit model includes two random parameters in the final model: one-way cost and waiting time. For these two parameters, the standard deviations of the lognormal distribution are significantly different from zero, as shown in Table 4.

**Table 4:** Distribution of the random parameters.

Random parameter	Distribution	Standard deviation	z-statistics	p-value
One-way cost ($)	Lognormal	1.268	14.56	<0.05
Waiting time (Minutes)	Lognormal	0.549	7.65	<0.05

As shown in Table 5, the random-parameter ordered Logit model identifies a number of socio-economic factors and trip characteristics that contribute to the heterogeneities in the influence of one-way cost and waiting time on perceived satisfaction with train fare.

**Table 5:** Heterogeneities in the random parameters.

Random Parameter	Attribute	Coefficient Estimate	z-statistics	p-value
One-way cost ($)	Household composition: Adults and children	-0.464	-4.13	<0.05
	Transport mode from home to the train station: Dropped-off	-0.727	-3.69	<0.05
	Transport mode from home to the train station: Driving	-0.485	-3.63	<0.05
Waiting time (Minutes)	Employment status: Self-employed	0.619	3.59	<0.05
	Transport mode from home to the train station: Driving	-0.345	-2.17	<0.05
	Trip purpose: Shopping/Personal/ Business	-0.364	-2.35	<0.05
	Paid fare status: Full fare	0.615	4.66	<0.05
	Train services’ influence on the current home location decision: Significant	-0.604	-4.27	<0.05
	Train services’ influence on the current home location decision: Moderate	-0.747	-3.53	<0.05

### Key socio-economic factors

The best fitted random-parameter ordered Logit model is estimated by the socio-economic factors discussed below.

#### (a) Gender

Female respondents are significantly (p-value < 0.05) less satisfied with their train fare compared with their male counterparts. When other factors are controlled, a female respondent’s estimated odds of responding “extremely satisfied”, rather than “satisfied”, “extremely dissatisfied”, “dissatisfied”, or “neutral”, decrease by 25% in comparison to a male respondent.

This finding is in line with gender differences in general. Women tend to be more sensitive to monetary costs, more likely to shop for higher quality products or services than men, and more effective in distributing their income [76, 77]. Dwyer [76] also found that women allocate most of their income to buying goods rather than procuring services, and allocate a smaller amount to their travel budgets. Consequently, women are more likely to have a higher expectation of train services for the fare paid compared with their male counterparts.

In addition, Ellaway et al. [42] found that male travellers prefer to use private vehicles, and are most likely to have alternatives to public transport [43, 78]. This implies that they tend to ride trains less often and have less familiarity with train services when compared with female travellers. Thus, male travellers tend to be more accepting of the asking fare, and do not have the same high expectation of train services as female travellers.

#### (b) City of origin

Table 3 shows that respondents’ perceived satisfaction with train fare differs significantly across cities. When other factors are controlled, Sydney, Melbourne, and Brisbane respondents feel less satisfied with train fare compared with respondents in Adelaide. Specifically, by holding other factors constant, when compared with a respondent from Adelaide, the estimated odds of a Sydney respondent responding that they were “extremely satisfied”—rather than “satisfied”, “extremely dissatisfied”, “dissatisfied”, or “neutral”—decrease by 30% (i.e., (1-exp(-0.357)) *100%) [74]. Similarly, the estimated odds of a Melbourne respondent responding that they were “extremely satisfied”—rather than “satisfied”, “extremely dissatisfied”, “dissatisfied”, or “neutral”—decrease by 34%. Also, for a respondent from Brisbane, these estimated odds decrease by 42%.

However, when other factors are controlled, compared with respondents from Adelaide, Perth respondents feel more satisfied with their train fare. Specifically, when controlling for other factors, when compared with a respondent from Adelaide, a Perth respondent’s estimated odds of responding that they are “extremely satisfied”, rather than “satisfied”, “extremely dissatisfied”, “dissatisfied”, or “neutral”, increase by 49% (i.e., (exp(0.398)-1)*100%) [74].

These findings are not surprising, given the different characteristics of the public transport network and the diverse train fare structures across the five cities as elaborated in the Context: train fare structures in the five Australian cities section. This issue is further discussed in the Intercity comparison section.

### Key trip characteristics

Our modelling results also clearly show that characteristics of their most recent train trip significantly influence respondents’ satisfaction with the fare for that trip, as elaborated below.

#### (a) Transport mode from home to the train station

Respondents who take the bus to the train station are significantly (p-value < 0.05) more satisfied with their paid train fare than other respondents. Specifically, when controlling for other factors, the estimated odds of an “extremely satisfied” response for someone who takes the bus rather than other transport modes to the station—rather than a “satisfied”, “extremely dissatisfied”, “dissatisfied”, or “neutral” response—increase by 50%.

Again, this finding is not surprising, as the importance of train station access is widely acknowledged in the literature [30, 38, 79, 80]. First, the transport mode from home to the station influences how much effort travellers need to exert and, also, the travel cost. This latter factor is especially vital in this study because of the availability of fare transfer between public transport trips within a certain time window in Sydney, Melbourne, Brisbane, Adelaide, and Perth. This transferability often leads to a reduced train fare for respondents who take the bus to the station [49–53]. A respondent’s experience with their mode of station access is able to influence their satisfaction with the train trip in general, and the paid train fare in particular [30]. The existence of advance transit systems in major train stations in Sydney, Melbourne, Brisbane, Adelaide, and Perth enables an easy transfer between bus and train. Therefore, as revealed in our analysis, bus access to the train station appears to positively contribute to a respondent’s satisfaction with their train fare.

#### (b) Concession fare

Eligibility for a concession fare significantly (p-value < 0.05) affects a respondent’s satisfaction with their train fare. Respondents who are eligible for a student concession fare feel less satisfied with train fare than respondents who are ineligible. When other factors are controlled, the estimated odds of an “extremely satisfied” response for a respondent who is eligible for student concession fare—rather than a “satisfied”, “extremely dissatisfied”, “dissatisfied”, or “neutral” response—decrease by 35% in comparison to a respondent who is ineligible. However, compared with respondents that are not eligible for any concession fare, respondents who are eligible for a senior concession fare feel more satisfied with their fare. Specifically, when controlling for other factors, compared with a respondent who is not eligible for any concessional fare, the estimated odds of an “extremely satisfied” response from a respondent who is eligible for a senior concession fare—rather than a “satisfied”, “extremely dissatisfied”, “dissatisfied”, or “neutral” response—increase by 10.6 times.

Student and senior concession fare are designed to provide subsidies for students and senior citizens respectively. The availability of subsidies influences seniors’ and student travellers’ perceived satisfaction with train fare. Respondents who are eligible for a senior concession fare pay less than respondents who are ineligible for any concession fare. Having received a subsidy through a reduced train fare, senior travellers feel more satisfied with their paid train fare. Subsidies are also directed to the disadvantaged transport group [81] who have decreased mobility, earn a low income, or both.

Interestingly, this study found that respondents who are eligible for student concession fares feel less satisfied with their train fare, despite having received a subsidy and only paying part of the adult non- concessional fare. This might simply imply that the student concession fare in the five targeted Australian cities is still perceived as too expensive by most students, who have no regular income and a limited travel budget.

#### (c) One-way cost

Not surprisingly, the one-way cost paid by each respondent significantly (p-value < 0.05) influenced his/her perceived satisfaction with train fare. Specifically, this study identified heterogeneity in the influence of one-way cost on the perceived satisfaction with train fare over the sampled population. The parameter of one-way cost varies significantly across respondents, as indicated by the significant standard deviation (p-value < 0.05) of its parameter. This finding is in line with earlier studies [40, 82, 83]. The marginal utility of one-way cost is linked with both socio-economic factors (household composition) and trip characteristics (i.e., transport mode from home to the train station). Further discussion on the heterogeneity of one-way cost is provided in the Heterogeneity sub-section.

#### (d) Waiting time

This study found that the impact of waiting time varies across respondents, as indicated by the significant (p-value < 0.05) standard deviation of its parameter. Table 5 shows how the perception of waiting time differs according to employment status, transport mode from home to the train station, trip purpose, paid fare status, and the influence of train services on the current home location. This finding is not surprising because the waiting time is perceived as an unproductive period, and travellers (who often experience long waiting times) feel less satisfied with public transport service and, consequently, more stressed than their counterparts [13, 31, 32, 39]. Long waiting times are caused by services not running to schedule. This lack of reliability of public transport results in travellers feeling a diminished sense of control [31]. Over time, long waiting times also lead to intense and prolonged feelings of stress. On the other hand, an increase on-board accessibility leads to both an increase in the train ridership increment, and a perceived satisfaction with the paid train fare [30, 84].

The heterogeneity of waiting time is also not surprising because the perception of waiting time can be influenced by numerous factors. For example, the availability of at-stop, real-time information and a comfortable waiting area can improve the perceived quality of public transport services by reducing the perceived waiting time [39, 85]. The availability of real-time information also reduces the unit costs of waiting time because travellers experience a more organized trip and reduced stress [85]. The marginal utility of one-way cost and further discussion on heterogeneity of waiting time is provided in the Heterogeneity sub-section.

### Interaction variables

The significant (p-value < 0.05) key interaction variables identified in the best-fitted ordered Logit model and how they affect the respondents’ level of satisfaction with train fare are discussed below.

#### (a) Sydney and employment status: Outside work force

There is a positive relationship between interaction of Sydney and outside work force against the level of satisfaction with train fare. By controlling for other factors, the estimated odds of an outside work force Sydney respondent responding that they were “extremely satisfied”—rather than “satisfied”, “extremely dissatisfied”, “dissatisfied”, or “neutral”—increase by 1.07 times (i.e., (exp(0.608+0.118)-1) *100%), compared with a respondent who is from Sydney but is not outside work force. In addition, regardless of the city, the outside work force employment status is positively related to a respondent’s satisfaction level with the paid train fare.

The aforementioned finding might be related to the impact of employment status on trip frequency and travel budget allocation [86]. Travellers’ perceptions of their trip is an important determinant of their satisfaction level [13], and the frequent and regular train rides of employed commuters enable them to establish a sense of familiarity with, and expectations of services. Not surprisingly, regular riders tend to have high expectations of train services for the fare they paid. However, outside workforce respondents’ train travel is generally less frequent and more irregular [79]. Thus, they are more likely to be less familiar with, and have lower expectations of train services. In addition, they are more likely to be satisfied with the fare paid for their most recent trip.

#### (b) Perth and transport mode from home to the train station: Bus

Interaction of Perth and taking bus to train station is negatively related with the level of satisfaction with train fare. This is quite interesting because such interaction makes taking bus from home to train station impact differently on the level of satisfaction with the paid train fare for a respondent from Perth, compared with that for a respondent from the other cities. More specifically, for a Perth respondent who takes bus from home to the train station, by controlling for other factors, the estimated odds of being “extremely satisfied”—rather than “satisfied”, “extremely dissatisfied”, “dissatisfied”, or “neutral”—decrease approximately by 8%, compared with a respondent who is from the same city but uses other modes for travelling from home to the train station. In contrast, for a respondent from any other city who takes bus from home to the train station, by controlling for other factors, the estimated odds of being “extremely satisfied”—rather than “satisfied”, “extremely dissatisfied”, “dissatisfied”, or “neutral”—increase by about 50%, compared with a respondent who is from the same city but uses other modes for travelling from home to the train station.

The finding above highlights the importance of the supporting role of bus service. The poor quality of the bus service that is part of a train trip can significantly and negatively impact a train user’s satisfaction with the train fare paid. It appears that Perth respondents are less pleased with the reliability of bus services to connect their homes to the nearest train stations than other cities’ respondents. It is possible that the bus stops are not evenly located across the surrounding neighbourhood or the schedules’ are not properly aligned with train services to cater the train riders’ demands.

#### (c) Sydney and student concession fare

A negative relationship is found between the interaction of Sydney and Student concession fare and the level of satisfaction with train fare. This demonstrates that students in Sydney have less appreciation towards student concession fare offered by Transport for New South Wales (NSW). The Transport for NSW official website mentions that student concession fare is only provided for primary and secondary students in NSW, full-time Australian tertiary students and limited full-time international students who are fully funded by specified Australian Government scholarships [49]. The best-fitted model perhaps reflects the scenario that many Sydney respondents who are 16–30 years old and are student do not actually meet the eligibility requirements for receiving the student concession fare.

#### (d) City of origin and senior concession fare

Our modelling analysis also reveals some complex interactions between city of origin and senior concession fare in terms of respondents’ satisfaction level with the paid train fare. Although receiving senior concession fare generally increases a respondent’s level of satisfaction with the paid train fare, this trend can be significantly influenced by respondents’ city of origin. For example, for a Melbourne respondent who receives the senior concession fare, by controlling for other factors, the estimated odds of being “extremely satisfied”—rather than “satisfied”, “extremely dissatisfied”, “dissatisfied”, or “neutral”—increase approximately by 2 times, compared with a Melbourne respondent who is not eligible for receiving the senior concession fare. A similar impact of receiving the senior concession fare is found for respondents from Brisbane as the estimated odds for a Brisbane respondent of being “extremely satisfied”—rather than “satisfied”, “extremely dissatisfied”, “dissatisfied”, or “neutral”—increase approximately by 2.9 times, by controlling for other factors. The positive impact of receiving the senior concession fare in the other cities are even stronger.

These findings establish that Melbourne and Brisbane respondents who are 60 or more years old and are retired appear to have less appreciation towards the senior concession fare offered by Victoria State Government [50] and by Queensland Government [51], respectively. This could be due to different eligibility criteria, such as minimum age limit, minimum paid working hours, and residency status set by transport authorities in each state [49–53]. Our model may have reflected the scenario that many Melbourne and Brisbane respondents, who are at least 60 years old and are retired, are not actually eligible for the senior concession fare in their respective city of origin.

### Heterogeneity

As discussed in the previous sub-sections, notable heterogeneity is detected across respondents in their perceived satisfaction with train fare. Specifically, significant heterogeneity is observed for one-way cost and waiting time. The analysis shows that this heterogeneity can be explained, to a certain extent, by socio-economic factors and trip characteristics, as elaborated below.

#### (a) Household composition

**Fig 2:**
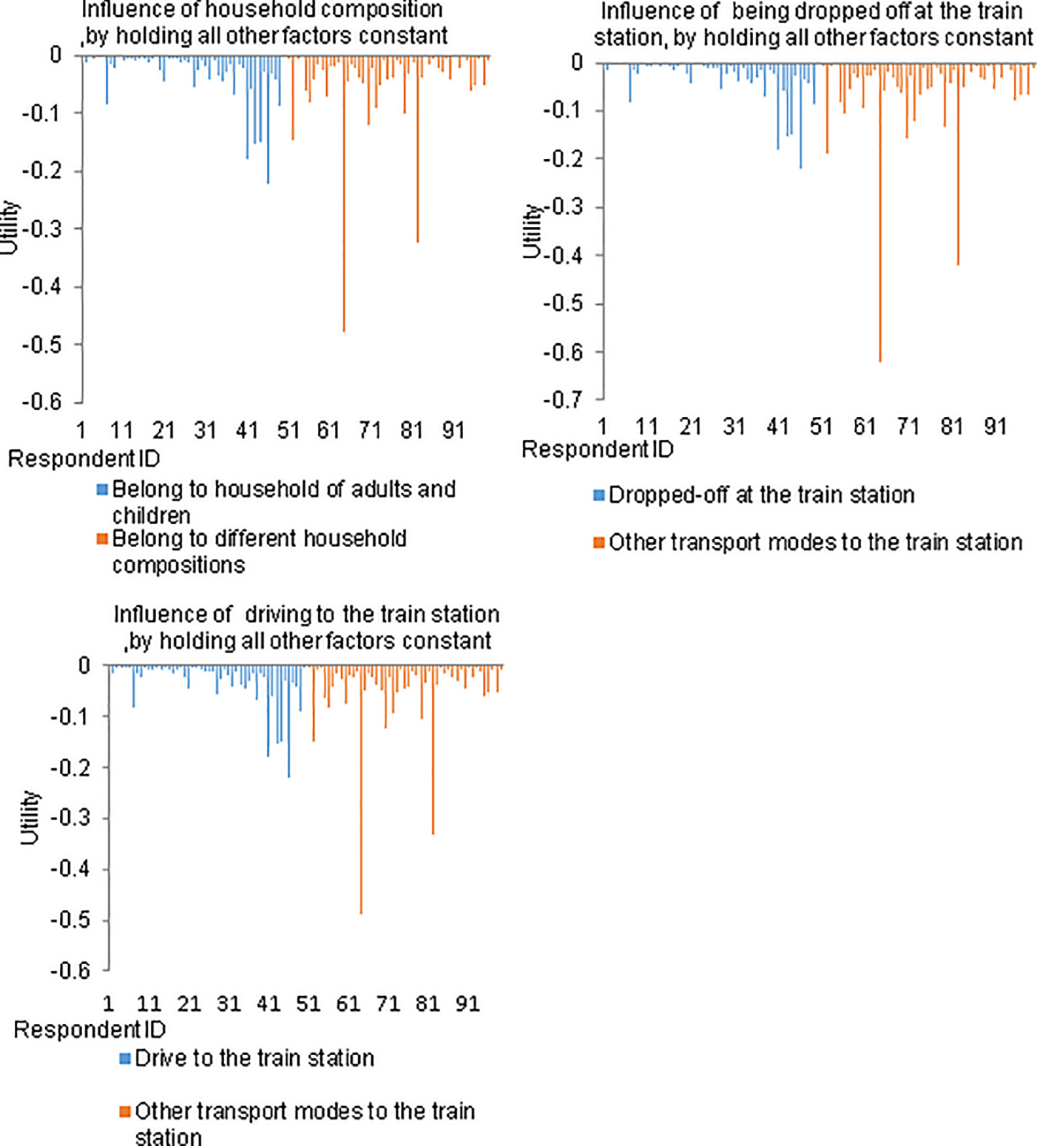
The simulated marginal utility of one-way cost for 100 randomly selected individuals.

Respondents who belong to the household of adults and children have a significant influence on the heterogeneity in respondents’ sensitivity to one-way costs. The marginal utility of one-way cost is:—*exp [-2*.*482–0*.*464 x (household of adults and children) -0*.*727 x (being dropped-off at the train station) -0*.*485 x (drive to the train station) + 1*.*268 x n]*, where *n* is a random number generated from a standard normal distribution. To gain insights into the influence of household composition on respondents’ sensitivity to one-way cost, this marginal utility has been simulated for 100 randomly selected individuals by holding both the transport modes from home to the train station factors constant, as shown in the top-left subfigure of Fig 2. Specifically, 50% of the randomly selected individuals belong to a household of adults and children. This figure clearly shows strong heterogeneity across individuals, regardless of their household composition.

By controlling randomness and other factors, individuals who belong to a household of adults and children are less sensitive to one-way cost than individuals from other type of households. Although individuals who belong to household of adults and children are likely to pay a large amount of train fare when travelling as a family, a heavily discounted fare is available for the children. On weekdays, the children fare is a small portion of the adult fare and, under certain circumstances, is free during weekends [49–53]. As a result, this group of individuals is less sensitive to the variation in one-way cost than their counterparts.

#### (b) Transport mode from home to the train station

Similarly, two other simulation exercises of marginal utility of one-way cost are performed for the mode to the train station variables, as shown in the top right and the bottom subfigures of Fig 2. Each simulation run aims to attain a deeper understanding on the influence of a particular key variable on the marginal utility of one-way cost, by controlling for the rest of variables. Each simulation specifies that half of the randomly selected individuals belong to a key variable in focus and the other half do not. In both simulations, clear heterogeneities across individuals were detected, regardless of their mode of transport from home to the station. Specifically, travellers who were dropped-off at, and who drove to the train station appear to be less sensitive to one-way cost than other travellers. This is probably because they are able to save money on fuel, parking, and toll costs by taking the train for the rest of their trip. They are also able to reduce their access time by driving to or being dropped off at the station instead of taking other modes of transport.

**Fig 3:**
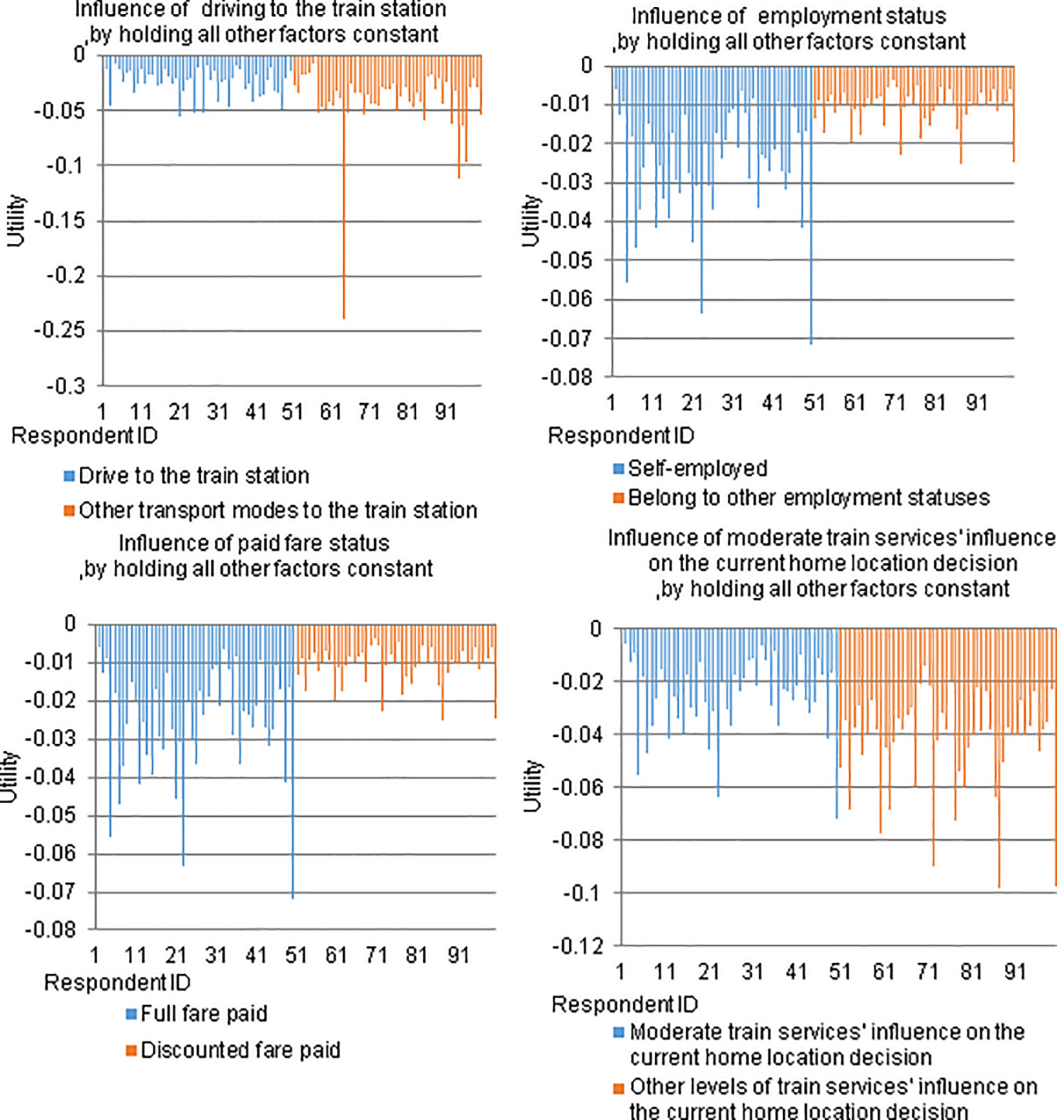
The simulated marginal utility of waiting time for 100 randomly selected individuals.

The marginal utility for waiting time is:—*exp [-2*.*988+ 0*.*619 x (self-employed)– 0*.*345 x (drive to the train station) -0*.*364 x (shopping/personal business trip) + 0*.*615 x (full fare) - 0*.*604 x (significant influence of train services on the current home location decision)– 0*.*747 x (moderate influence of train services on the current home location decision) + 0*.*549 x n]*, where *n* is a random number generated from a standard normal distribution. Like the previous one, this marginal utility has been simulated for 100 randomly selected individuals as shown in the top-left subfigure of Fig 3. Specifically, half of the randomly selected individuals are driving to the station. This study finds travellers who drove to the station are less sensitive to waiting time compared to other travellers. This is consistent with our everyday experience because driving to the station often gives a traveller more control over their departure time and they are more likely to arrive at the station as scheduled to endure less waiting time.

Similarly, another five simulations of the marginal utility of waiting time are performed for the rest key variables (three of them are presented in Fig 3 for illustration purpose), and notable heterogeneities of marginal utility values are also found in the simulation results, as elaborated below.

#### (c) Employment status

By controlling randomness and other factors, the marginal disutility values of waiting time of self-employed individuals are much larger than respondents with other employment status. This finding is not surprising because it is in self-employed respondents’ best interest to be time-conscious so as not to disadvantage business; hence, they tend to be very sensitive to waiting time. In addition, as shown in the top-right subfigure of Fig 3, regardless of the employment status, significant heterogeneities exist across individuals.

#### (d) Trip purpose

When randomness and other factors are controlled, the marginal disutility values of waiting time of individuals whose trips are for shopping or personal business purposes are smaller than those of individuals whose trips are for other purposes. Generally, the group of individuals who are travelling for shopping or personal business purposes have irregular travel patterns. Hence, they tend not to be sensitive to fluctuations in waiting time.

#### (e) Paid fare status

The marginal utility function for waiting time also indicates that by controlling randomness and other factors, individuals who pay full fare and travel during peak hours are generally more sensitive to waiting time than individuals who pay a discounted fare and travel during off-peak hours. This finding is also consistent with our daily experience. As a trade-off for paying full fare and experiencing on-board crowding, peak hour travellers are more likely to expect the advantage of higher frequency train services compared with travelling off-peak. Consequently, they tend to be more sensitive to waiting for their targeted train services. In addition, as shown in the bottom-left subfigure of Fig 3, significant heterogeneities exist across individuals, regardless of the amount of fare they pay.

#### (f) Influence of train services on the current home location decision

As shown in the bottom-right subfigure of Fig 3, the moderate and significant influences of train services on the current home location decision also contribute to the explanation of preference heterogeneity detected in the marginal utility for waiting time. This marginal utility function reveals that when randomness and other factors are controlled, the marginal disutility values of individuals who are moderately influenced by train services on the current home location decision are smaller than those of their counterparts. Similar trend has been found on the marginal disutility values of individuals who are significantly influenced by train services on the current home location decision are smaller in comparison to those of their counterparts.

These findings are both interesting and somewhat surprising. Respondents who are moderately and significantly influenced by train services in making their current home location decision would be expected to be sensitive to waiting time [87]. Nevertheless, this study finds quite the opposite. It seems that once this group of respondents have considered train services in making their current home location decision, they tend to accept variations in waiting time.

## Intercity comparison

“Context: train fare structures in the five Australian cities” section has described the current fare structure in each city, which reveals notable differences in how train fares are structured across these five cities. More specifically, train fares in Sydney, Melbourne, and Brisbane rise as the number of zones or distance travelled increase. Our data show that a number of respondents from Sydney, Melbourne, and Brisbane travelled about 15 km during their most recent home-based train trip. In Sydney, Melbourne, and Brisbane, public transport travellers respectively pay $4.20, $3.90, and $5.96 for a one-way, 15 km trip during peak hours on any weekday [49–51]. The regular public transport fare in Adelaide for a single trip using MetroCard is $ 3.54, regardless of the distance travelled [53]. According to our data, the structure of Adelaide’s public transport fares benefits most Adelaide respondents whose trip origins are within 15 km of their destinations. For the same travelled distance, Adelaide respondents pay the least one-way fare. Consequently, Adelaide respondents feel more satisfied with their train fares than Sydney, Melbourne, and Brisbane respondents.

Meanwhile, when comparing Perth and Adelaide, the fact that many Adelaide respondents are required to pay a fixed fare regardless of the distance travelled can negatively impact their satisfaction with their train fare. In Perth, on the other hand, public transport fares are determined by the number of zones travelled. According to the survey responses, Perth respondents’ origins for their most recent train trips were often located around 16 km from their destinations. Since one public transport zone in Perth is approximately 8 km in radius, the respondents commute daily for at least two zones one-way, and pay at least $ 3.91 for a regular SmartRider fare during peak periods [52]. In contrast, Adelaide respondents who travel a short distance from home on a train can pay the same fare as those who travel a long distance. Therefore, Adelaide respondents tend to be less contented with their train fares than Perth respondents.

## Conclusions

Based on a nationwide survey, this study focused on train riders’ satisfaction with the fare they paid for their most recent trip. The influence of their socio-economic profiles and their specific trip characteristics on these perceived satisfactions are assessed and quantified using an ordered Logit model.

This study identified that train riders’ socio-economic profiles can significantly influence their satisfaction with their train fare. Specifically, female respondents tend to be less satisfied with the fare than their male counterparts. In addition, respondents’ perceived satisfaction with train fare significantly differs across cities. When other factors are controlled, Sydney, Melbourne and Brisbane respondents feel less satisfied with train fare than Adelaide respondents. To attain a deeper knowledge, modelling results are discussed in the context of the different train fare structures in the five cities. Specifically, for the same travelled distance, Adelaide respondents pay the least for a one-way trip compared with Sydney, Melbourne, and Brisbane respondents. On the contrary, Perth respondents are likely to be more contented with their train fares than Adelaide respondents.

Around one fifth of respondents in Sydney, Melbourne, Adelaide, and Perth did not report their income level, while about 15% of respondents in Brisbane chose not to report their income level. Respondents’ reluctance of reporting their income is a widely acknowledged challenge in the survey literature [88, 89]. Such reluctance’s impact on the data analysis can be very complex. The literature suggests that people who are not willing to disclose their income tend to feel more insecure, and are consequently more sensitive to monetary costs [90–92]. It would be interesting to test and differentiate high and low non-reported income groups’ effect. Unfortunately, by the very nature of being not reported, it would be very difficult to categorize a non-reported income into a high or low income group, which makes it almost impossible to detect and scrutinize any phenomenon caused by different non-reported incomes. In this study, the ‘Not reported income’ variable has been found not to be statistically significant in the best-fitted ordered logit model.

Meanwhile, characteristics of the train trip also significantly influence a rider’s satisfaction with their train fare. Respondents’ perceived satisfaction is significantly impacted by station access and their eligibility for a concession fare. Respondents who take the bus to the train station appeared to be more contented with their paid train fares in comparison to other respondents. Nonetheless, it is not the case for Perth respondents. A Perth respondent who takes bus from home to the train station tend to be less satisfied compared with a respondent who is from the same city but uses other modes to reach the train station. It is possible that the bus services in Perth are not seamlessly connected with the train stations.

Respondents who are eligible for a student concession fare feel less satisfied with train fares than ineligible respondents. Specifically, a negative relationship is found between the interaction of Sydney and Student concession fare and the level of satisfaction with train fare. Sydney respondents who are 16–30 years old and are students tend to have a low appreciation towards the concession fare offered by Transport for New South Wales (NSW) [49]. It may be due to strict eligibility rules imposed by Transport for New South Wales (NSW). Conversely, respondents who are eligible for a senior concession fare feel more pleased with their train fares compared with respondents that are not eligible for any concession fare.

Having taken into account findings in previous literature [55, 93], on-board crowding is considered as a random-parameter at the start of model estimation. However, this variable turns out to be not statistically significant in the best-fitted ordered logit model. This result may be caused by the potential confounding effect of the self-reported crowdedness because the self-reported crowdedness can be inconsistent with the actual crowdedness. Future studies could use some objective measure crowdedness (e.g., number of passengers) into the train fare satisfaction model developed in this study.

Moreover, notable heterogeneity in their perceived satisfaction with train fare was detected across respondents. Specifically, one-way cost and waiting time were found to be the significant random parameters. The marginal utility of one-way cost is linked with both socio-economic factors and trip characteristics. Household composition and station access were found to have a significant influence on heterogeneity in respondents’ sensitivity to one-way cost. Meanwhile, the disutility value of waiting time varies significantly across respondents, and is influenced by their employment status, transport mode from home to the station, trip purpose, paid fare status, and the influence of train services on their current home location.

A number of earlier studies mentioned the important role of pre-departure information. This information allows travellers to plan their trip in advance, and provides a basis for their loyalty to a particular transport mode [31, 37–40]. Having obtained their trip information, travellers can anticipate a straightforward trip, as well as the ability to make route changes should the unexpected occur [37]. However, this study found that once they had chosen the train as their transport mode, respondents’ perceived satisfaction with their train fare was not significantly influenced by whether they had checked pre-departure information or not. Despite the apparent demand for the provision of complete trip information at train stations, there is still a need to scientifically examine the impact of the availability, reliability, and usefulness of such information on train ridership and traveller satisfaction [41]. This is especially the case, given that the provision of unreliable trip information can be a major source of decline in train ridership [31].

The importance and urgency of determining factors that influence travellers’ perceived satisfaction with train fares is frequently recognized in the literature [13, 30, 42, 46, 47], as such knowledge is critical for policy makers and transport operators in developing effective future transit policies. In this regard, the findings of this study are significant and useful. On-going efforts in this research area need to determine the influence of specific socio-economic factors and trip characteristics on travellers’ choice of transport mode. It will be useful, for example, to determine whether the key variables found in this study affect respondents’ preference for a particular transport mode, and their loyalty to that mode. The stated-preference data collected in this survey can assist in this further investigation.

## Supporting information

S1 Dataset (XLSX)
